# Artificial intelligence-enabled electrocardiogram model for predicting heart failure with preserved ejection fraction: a single-center study

**DOI:** 10.1093/ehjdh/ztaf080

**Published:** 2025-07-17

**Authors:** David Hong, Sung-Hee Song, Heayoung Shin, Minjung Bak, Juwon Kim, Darae Kim, Ju Youn Kim, Jeong Hoon Yang, Seung-Jung Park, Jin-Oh Choi, Young Keun On, Kyoung-Min Park

**Affiliations:** Division of Cardiology, Department of Medicine, Heart Vascular Stroke Institute, Samsung Medical Center, Sungkyunkwan University School of Medicine, 81 Irwon-ro, Gangnam-gu, Seoul 06351, Republic of Korea; Wellysis Corp., Seoul, Republic of Korea; Division of Cardiology, Department of Medicine, Heart Vascular Stroke Institute, Samsung Medical Center, Sungkyunkwan University School of Medicine, 81 Irwon-ro, Gangnam-gu, Seoul 06351, Republic of Korea; Division of Cardiology, Department of Medicine, Heart Vascular Stroke Institute, Samsung Medical Center, Sungkyunkwan University School of Medicine, 81 Irwon-ro, Gangnam-gu, Seoul 06351, Republic of Korea; Division of Cardiology, Department of Internal Medicine, Korea University Guro Hospital, Korea University College of Medicine, Seoul, Republic of Korea; Division of Cardiology, Department of Medicine, Heart Vascular Stroke Institute, Samsung Medical Center, Sungkyunkwan University School of Medicine, 81 Irwon-ro, Gangnam-gu, Seoul 06351, Republic of Korea; Division of Cardiology, Department of Medicine, Heart Vascular Stroke Institute, Samsung Medical Center, Sungkyunkwan University School of Medicine, 81 Irwon-ro, Gangnam-gu, Seoul 06351, Republic of Korea; Division of Cardiology, Department of Medicine, Heart Vascular Stroke Institute, Samsung Medical Center, Sungkyunkwan University School of Medicine, 81 Irwon-ro, Gangnam-gu, Seoul 06351, Republic of Korea; Division of Cardiology, Department of Medicine, Heart Vascular Stroke Institute, Samsung Medical Center, Sungkyunkwan University School of Medicine, 81 Irwon-ro, Gangnam-gu, Seoul 06351, Republic of Korea; Division of Cardiology, Department of Medicine, Heart Vascular Stroke Institute, Samsung Medical Center, Sungkyunkwan University School of Medicine, 81 Irwon-ro, Gangnam-gu, Seoul 06351, Republic of Korea; Division of Cardiology, Department of Medicine, Heart Vascular Stroke Institute, Samsung Medical Center, Sungkyunkwan University School of Medicine, 81 Irwon-ro, Gangnam-gu, Seoul 06351, Republic of Korea; Division of Cardiology, Department of Medicine, Heart Vascular Stroke Institute, Samsung Medical Center, Sungkyunkwan University School of Medicine, 81 Irwon-ro, Gangnam-gu, Seoul 06351, Republic of Korea; Division of Cardiology, Department of Medicine, Heart Vascular Stroke Institute, Samsung Medical Center, Sungkyunkwan University School of Medicine, 81 Irwon-ro, Gangnam-gu, Seoul 06351, Republic of Korea

**Keywords:** Artificial intelligence, Electrocardiogram, Heart failure

## Abstract

**Aims:**

Heart failure with preserved ejection fraction (HFpEF) is difficult to diagnose due to the lack of a definitive diagnostic marker; multiple tests are required, including advanced evaluations. This study aimed to develop an artificial intelligence (AI)-enabled electrocardiogram (ECG) model for predicting HFpEF.

**Methods and results:**

This retrospective cohort study included patients from a single tertiary centre who underwent echocardiography, N-terminal prohormone of B-type natriuretic peptide measurement, and ECG within a defined timeframe. Patients were classified as HFpEF (HFA-PEFF score ≥5) or control (HFA-PEFF score <5). Patients were divided into training, validation, and test subsets at a 7:1:2 ratio for model development and validation. Using the collected ECGs, a convolutional neural network was trained to predict HFpEF; its performance was assessed using the area under the receiver operating characteristic curve (AUROC). Among the 13 081 patients included, 5795 (44.3%) were classified as HFpEF and 7286 (55.7%) were classified as control. The AI-enabled ECG model demonstrated good discriminative performance [AUROC 0.81; 95% confidence interval (CI) 0.79–0.82]. Subgroup analyses stratified by HFpEF risk factors confirmed consistent model performance. Prognostic evaluation revealed that patients with a positive AI-ECG classification experienced significantly worse outcomes relative to those with a negative classification, including higher risks of cardiac death (1.1% vs. 0.1%; hazard ratio 9.56; 95% CI 1.24–73.53; *P* = 0.030) and heart failure hospitalization (2.8% vs. 0.6%; hazard ratio 5.91; 95% CI 2.08–16.81; *P* = 0.001) at 5 year.

**Conclusion:**

The AI-ECG model is a reliable tool for predicting HFpEF, as defined by the HFA-PEFF score, and effectively stratifies patients according to prognosis. Integration of this model into clinical practice may simplify and enhance the diagnostic process for HFpEF.

## Graphical Abstract

**Graphical Abstract:**
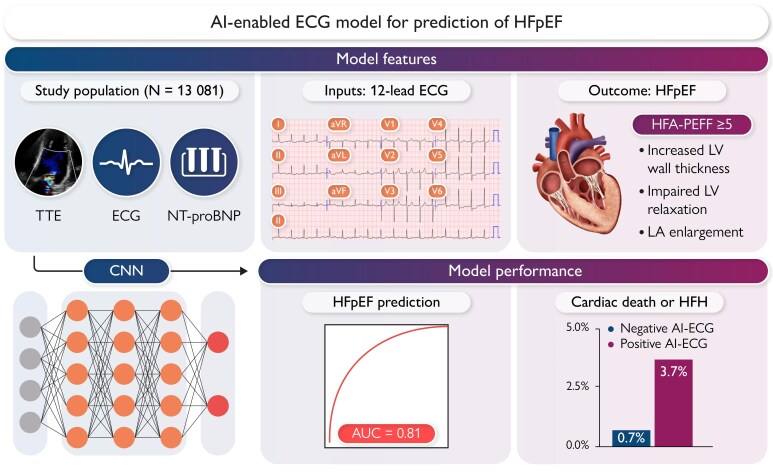
AI, artificial intelligence; AUC, area under the curve; CNN, convolutional neural network; ECG, electrocardiogram; HFA-PEFF, The Heart Failure Association Pre-test Assessment, Echocardiography and natriuretic peptide, Functional testing, and Final Etiology; HFH, heart failure hospitalization; HFpEF, heart failure with preserved ejection fraction; LA, left atrial; LV, left ventricular; NT-proBNP, N-terminal prohormone of B-type natriuretic peptide; TTE, transthoracic echocardiography.

## Introduction

Heart failure (HF) with preserved ejection fraction (HFpEF) represents a growing health challenge within ageing populations, causing nearly half of all HF cases.^1^ HFpEF is associated with substantial morbidity and mortality, emphasizing the need for timely and accurate diagnosis to improve patient outcomes.^2,3^ Unlike HF with reduced ejection fraction, where a reduced left ventricular ejection fraction serves as a definitive diagnostic criterion, the diagnosis of HFpEF lacks a single, definitive marker and requires a structured, multimodal approach. This process involves evaluating multiple echocardiographic parameters to assess diastolic dysfunction and structural changes, along with the measurement of biomarkers such as N-terminal prohormone of B-type natriuretic peptide (NT-proBNP).^4^ The Heart Failure Association Pre-test Assessment, Echocardiography and natriuretic peptide, Functional testing, and Final Etiology (HFA-PEFF) score integrates these diagnostic components into a standardized framework.^5^ However, this approach requires imaging specialists, advanced healthcare facilities, and substantial costs, making HFpEF diagnosis particularly challenging in routine clinical practice.^5,6^ These challenges underscore the need for innovative diagnostic tools that are accessible, reliable, and capable of aiding in HFpEF screening and early diagnosis.

Artificial intelligence (AI)-enabled tools, such as electrocardiogram (ECG) models, constitute a promising solution to these challenges. By leveraging routinely available ECG data, AI models can offer a cost-effective and widely accessible method for identifying patients with HFpEF. This study aimed to develop an AI-based ECG model to predict HFpEF, using the HFA-PEFF score as the reference standard,^5^ and assess its prognostic relevance.

## Methods

### Study population and design

**Figure 1:**
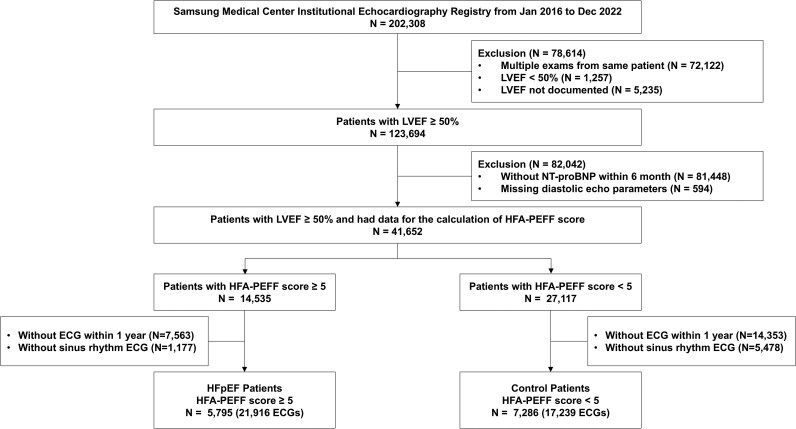
Study flow. This retrospective cohort study included patients who underwent echocardiography, N-terminal prohormone of B-type natriuretic peptide measurement, and electrocardiogram within a defined timeframe at Samsung Medical Center between January 2016 and December 2022. Patients were classified as HFpEF (Heart Failure Association Pre-test Assessment, Echocardiography and natriuretic peptide, Functional testing, and Final Etiology score ≥5) or control (Heart Failure Association Pre-test Assessment, Echocardiography and natriuretic peptide, Functional testing, and Final Etiology score <5). A convolutional neural network model was trained using the collected electrocardiograms to predict heart failure with preserved ejection fraction. ECG, electrocardiogram; HFA-PEFF, The Heart Failure Association Pre-test Assessment, Echocardiography and natriuretic peptide, Functional testing, and Final Etiology; HFpEF, heart failure with preserved ejection fraction; LVEF, left ventricular ejection fraction; NT-proBNP, N-terminal prohormone of B-type natriuretic peptide.

This retrospective cohort study included consecutive patients who underwent transthoracic echocardiography at Samsung Medical Center, South Korea, between January 2016 and December 2022. Patients were excluded if they met any of the following criteria: left ventricular ejection fraction <50%, absence of NT-proBNP measurements within 6 months before or after echocardiography, incomplete parameters required for calculation of the HFA-PEFF score during echocardiography, or lack of sinus rhythm ECGs recorded within 1 year before or after echocardiography (*Figure 1*).

The HFA-PEFF score was calculated for all included patients and used as the reference standard for HFpEF diagnosis, with a score ≥5 classified as HFpEF.^5^ This study aimed to develop an AI algorithm to predict HFpEF using sinus rhythm ECGs recorded before or after echocardiography (see Supplementary material online, *Figure S1*). The final cohort was divided into training, validation, and test subsets at a 7:1:2 ratio for model development and validation. Importantly, only the training and validation cohorts were utilized to develop the AI-enabled ECG model; the test cohort was exclusively reserved for validation of the developed model. For patients in the test cohort with multiple ECGs, the ECG closest to the time of echocardiography was selected as the representative for validation. The study protocol was approved by the Institutional Review Board of Samsung Medical Center (IRB No. 2020-01-007) and conducted in accordance with the principles outlined in the Declaration of Helsinki. The requirement for informed consent was waived by the Institutional Review Board due to the retrospective nature of the study and exclusive use of anonymized data.

### The Heart Failure Association Pre-test Assessment, Echocardiography and natriuretic peptide, Functional testing, and Final Etiology diagnostic algorithm

The HFA-PEFF diagnostic algorithm was developed to provide a systematic, accurate, and accessible framework for diagnosing HFpEF.^5^ Its diagnostic performance has been validated in various external cohorts.^7,8^ The algorithm follows a structured, stepwise approach and begins with a pre-test assessment to exclude non-cardiac causes and identify patients with suspected HFpEF. The next step involves diagnostic scoring based on echocardiographic findings and biomarker levels. To summarize its components and calculation method: the HFA-PEFF score includes three domains—functional, morphological, and biomarker. Patients are assigned a score of 0, 1 (minor), or 2 points (major) in each domain, and sub-scores are summed to yield a total score ranging from 0 to 6. The total score is classified into three likelihood levels: low (0–1 point), intermediate (2–4 points), and high (5–6 points). Patients with a low probability score are considered unlikely to have HFpEF, while those with a high probability score are diagnosed with HFpEF. For those with an intermediate probability, further advanced work-up, such as exercise echocardiography or invasive catheterization, is recommended to confirm or rule out the diagnosis.^5^

Transthoracic echocardiography, required to calculate the functional and morphological domains, was performed in accordance with contemporary guidelines.^9,10^ Interventricular septum thickness, posterior wall thickness, and left ventricular internal diameters during diastole and systole were measured using M-mode imaging in the parasternal long-axis view. Left ventricular ejection fraction was assessed via the biplane Simpson's method or M-mode imaging. Left ventricular end-diastolic and end-systolic volumes and left atrial volume were determined from apical four- and two-chamber views using the biplane method of discs. The left atrial volume index was calculated as left atrial volume divided by body surface area (mL/m^2^). Peak early and late diastolic mitral flow velocities (E and A) were measured using pulsed-wave Doppler analysis. Peak early diastolic velocity of the mitral annulus (*e*′) was assessed at the septal aspect of the mitral annulus. Right ventricular systolic pressure was determined using peak tricuspid regurgitation velocity measured with continuous Doppler.

### Artificial intelligence-enabled electrocardiogram model development

All ECGs were recorded at a sampling rate of 500 Hz using Philips ECG instruments (PageWriter TC70, TC50, TC30, and Trim III) for standard 10-s 12-lead ECGs. Raw data were stored in extensible mark-up language format; an ECG database was constructed using the collected ECGs, each labelled with interpretations provided by trained physicians and cardiologists.

A one-dimensional adaptation of the DenseNet-121 convolutional neural network architecture was implemented to analyse ECG signals and capture subtle temporal features (see Supplementary material online, *Figure S2*).^11^ The network begins with an initial convolutional layer comprising 64 filters, a kernel size of 7, and strides of 2, followed by batch normalization and rectified linear unit activation. Max pooling is then applied, with a pool size of 3 and strides of 2, to downsample the input. The network proceeds with dense blocks, each beginning with a bottleneck convolutional layer that utilizes four times the number of input filters, a kernel size of 1, and strides of 1. This is followed by a feature extraction convolutional layer, which maintains the number of input filters and uses a kernel size of 3 to capture complex features. Outputs from these layers are concatenated within each block. Transition layers between dense blocks reduce the number of filters, utilizing a convolutional layer with half the input filters and average pooling with a pool size of 2 and strides of 2. These layers enable downsampling of the feature maps and reduce computational costs. This architecture, comprising initial, dense, and transition layers, is repeated to yield a total of 121 layers. Batch normalization and dropout are applied at each layer to enhance model performance and prevent overfitting. Pooling layers are used to downsample signals by reducing every two values to one, retaining the most relevant features while optimizing memory usage. Skip connections ensure dimensional consistency when merging paths at each block, facilitating improved information flow across the network. The proposed one-dimensional DenseNet-121 model was trained using labelled ECG data to identify features or patterns associated with HFpEF.

### Data collection and outcomes

The following data were retrospectively collected through a review of electronic medical records: demographic characteristics, comorbidities, laboratory findings, electrocardiographic findings, echocardiographic findings, and clinical outcomes during follow-up.

The primary outcome was the discriminative ability of the developed AI-enabled ECG model. Secondary outcomes comprised cardiac death, HF hospitalization, and the composite of both outcomes at 5 year. Cardiac death was defined as death due to cardiac disease; deaths of uncertain cause were excluded. Hospitalization for HF was defined based on the following criteria, which were slightly modified to accommodate the retrospective study design: (i) hospitalization with a primary diagnosis of HF, (ii) hospitalization duration of at least 12 h, (iii) new or worsening HF symptoms, (iv) objective evidence of new or worsening HF on laboratory findings, and (v) initiation or intensification of HF treatment.^12^

### Statistical analyses

Categorical variables are presented as numbers and relative frequencies (percentages) and were compared using the *χ*² test. Continuous variables are reported as means ± standard deviations or medians with interquartile ranges and were compared using Student's *t*-test or the Mann–Whitney rank-sum test, as appropriate. The discriminative ability of the model was evaluated through receiver operating characteristic curve analysis and area under the curve (AUC). Sensitivity, specificity, positive predictive value, negative predictive value, and diagnostic accuracy were calculated at the Youden index threshold. Sensitivity analyses were performed by including only individuals with clearly defined HFpEF status, excluding those at intermediate risk (HFA-PEFF score 2–4 points), and restricting the cohort to individuals who underwent an ECG within 3 months before or after echocardiography. Subgroup analysis was conducted based on age ≥65 years, body mass index ≥25 kg/m^2^, hypertension, and diabetes mellitus; discriminative ability between subgroups was compared using DeLong's test. Cumulative incidences of outcomes were assessed using Kaplan–Meier analyses. Cox proportional hazards regression models were utilized to compare the risk of clinical outcomes and to calculate hazard ratios (HRs) and 95% confidence intervals (CIs) between groups. The proportional hazards assumption was assessed using log-log survival function plots and Schoenfeld residuals. All probability values were two-sided, and *P*-values <0.05 were considered statistically significant. Statistical analyses were performed using R version 4.2.3 (R Foundation for Statistical Computing, Vienna, Austria) and Python version 3.8.

## Results

### Baseline characteristics of the study population

In total, 13 081 patients were included in this study based on the enrolment criteria (*Figure 1*). Among these patients, 5795 (44.3%) with an HFA-PEFF score ≥5 were classified as HFpEF, whereas 7286 (55.7%) with a score <5 were classified as controls. The cohort was divided into training (*N* = 9,156, 70%), validation (*N* = 1,308, 10%), and test (*N* = 2,617, 20%) subsets. The study population had a mean age of 63.6 years, and 54.4% of participants were men (*Table 1*). Compared with the control group, the HFpEF group was older, had a higher proportion of women, and exhibited a higher prevalence of comorbidities (e.g. hypertension, diabetes, atrial fibrillation, and chronic kidney disease). The HFpEF group also showed naturally elevated NT-proBNP levels and significant differences in functional and structural parameters, such as the left atrial volume index, the ratio of mitral flow velocity to early mitral annular velocity (*E*/*e*′), and right ventricular systolic pressure, all of which serve as components of the HFA-PEFF score. These differences were consistently observed between groups classified as positive or negative by the AI-enabled ECG model (*Table 2*). No significant differences in any parameters were noted when comparing the training, validation, and test cohorts (see Supplementary material online, *Table S1*).

**Table 1:** Baseline characteristics of study population

Variables	Total *N* = 13 081	Control *N* = 7286	HFpEF *N* = 5795	*P*-value
Age	63.6 ± 13.7	60.2 ± 13.8	67.9 ± 12.2	<0.001
Male	7121 (54.4)	4158 (57.1)	2963 (51.1)	<0.001
Systolic blood pressure, mmHg	125.8 ± 19.5	124.6 ± 18.2	127.4 ± 20.8	<0.001
Diastolic blood pressure, mmHg	71.2 ± 12.9	72.3 ± 12.5	69.8 ± 13.2	<0.001
Body mass index, kg/m^2^	24.4 ± 3.8	24.7 ± 3.7	24.0 ± 3.8	<0.001
Hypertension	8241 (63.0)	3998 (54.9)	4243 (73.2)	<0.001
Diabetes mellitus	4092 (31.3)	1898 (26.0)	2194 (37.9)	<0.001
Atrial fibrillation	317 (2.4)	2 (0.0)	315 (5.4)	<0.001
Chronic kidney disease	672 (5.1)	104 (1.4)	568 (9.8)	<0.001
Current or past smoking	3900 (29.8)	2190 (30.1)	1710 (29.5)	0.495
History of percutaneous coronary intervention	880 (6.7)	433 (5.9)	447 (7.7)	<0.001
History of myocardial infarction	543 (4.2)	222 (3.0)	321 (5.5)	<0.001
Chronic obstructive lung disease	713 (5.5)	364 (5.0)	349 (6.0)	0.010
N-terminal prohormone of B-type natriuretic peptide	172.0 [59.1–610.5]	66.8 [33.9–123.0]	621.1 [286.8–1811.5]	<0.001
Echocardiography				
Left ventricular internal diameter in diastole, mm	49.4 ± 5.6	48.9 ± 5.0	50.0 ± 6.3	<0.001
Left ventricular internal diameter in systole, mm	29.6 ± 4.7	29.0 ± 4.1	30.3 ± 5.3	<0.001
Left atrial volume index, mL/m^2^	39.8 ± 18.9	32.2 ± 9.8	49.3 ± 22.8	<0.001
Left ventricular mass index, g/m^2^	110.1 ± 35.8	99.3 ± 26.6	123.7 ± 40.9	<0.001
Peak early diastolic mitral flow velocity (*E*), m/s	0.71 ± 0.29	0.64 ± 0.19	0.79 ± 0.37	<0.001
Peak late diastolic mitral flow velocity (*A*), m/s	0.78 ± 0.25	0.73 ± 0.21	0.86 ± 0.29	<0.001
Early to late diastolic mitral flow velocities (*E*/*A*)	0.97 ± 0.89	0.96 ± 0.86	0.99 ± 0.92	0.039
Peak early diastolic velocity of mitral annulus (*e*′), m/s	0.07 ± 0.02	0.07 ± 0.02	0.06 ± 0.02	<0.001
Mitral flow velocity to early mitral annular velocity (E/e′)	11.8 ± 6.6	9.3 ± 3.3	15.0 ± 8.2	<0.001
Right ventricular systolic pressure, mmHg	30.0 ± 10.4	26.7 ± 6.8	33.7 ± 12.3	<0.001
Left ventricular ejection fraction, %	63.8 ± 6.1	64.3 ± 5.8	63.1 ± 6.3	<0.001
HFA-PEFF points				<0.001
0–1 (low probability)	1541 (11.8)	1541 (21.2)	0 (0)	
2–4 (intermediate probability)	5745 (43.9)	5745 (78.8)	0 (0)	
5–6 (high probability)	5795 (44.3)	0 (0)	5795 (100.0)	

Data are presented as the mean ± standard deviation, median [interquartile range], or *n* (%).

HFA-PEFF, Heart Failure Association Pre-test Assessment, Echocardiography and natriuretic peptide, Functional testing, Final Etiology; HFpEF, heart failure with preserved ejection fraction.

**Table 2:** Baseline characteristics according to artificial intelligence-enabled electrocardiogram model

Variables	Negative AI-ECG *N* = 1125	Positive AI-ECG *N* = 1492	*P*-value
Age	59.7 ± 13.4	67.1 ± 12.6	<0.001
Male	691 (61.4)	732 (49.1)	<0.001
Systolic blood pressure, mmHg	124.3 ± 18.4	127.4 ± 20.7	<0.001
Diastolic blood pressure, mmHg	72.4 ± 12.9	70.4 ± 13.2	<0.001
Body mass index, kg/m^2^	24.7 ± 3.6	24.0 ± 3.8	<0.001
Hypertension	638 (56.7)	1041 (69.8)	<0.001
Diabetes mellitus	304 (27.0)	538 (36.1)	<0.001
Atrial fibrillation	3 (0.3)	52 (3.5)	<0.001
Chronic kidney disease	32 (2.8)	101 (6.8)	<0.001
Current or past smoking	353 (31.4)	418 (28.0)	0.062
History of percutaneous coronary intervention	71 (6.3)	115 (7.7)	0.169
History of myocardial infarction	43 (3.8)	65 (4.4)	0.496
Chronic obstructive lung disease	56 (5.0)	96 (6.4)	0.115
N-terminal prohormone of B-type natriuretic peptide	75.5 [32.5–195.0]	356.3 [129.4–1168.5]	<0.001
Echocardiography			
Left ventricular internal diameter in diastole, mm	49.0 ± 5.0	49.5 ± 6.0	0.026
Left ventricular internal diameter in systole, mm	29.3 ± 4.1	29.8 ± 5.2	0.002
Left atrial volume index, mL/m^2^	33.1 ± 10.7	44.5 ± 25.0	<0.001
Left ventricular mass index, g/m^2^	100.1 ± 26.7	118.6 ± 42.8	<0.001
Peak early diastolic mitral flow velocity (*E*), m/s	0.65 ± 0.20	0.74 ± 0.33	<0.001
Peak late diastolic mitral flow velocity (*A*), m/s	0.72 ± 0.20	0.83 ± 0.27	<0.001
Early to late diastolic mitral flow velocities (*E*/*A*)	0.97 ± 0.48	0.95 ± 0.66	0.246
Peak early diastolic velocity of mitral annulus (*e*′), cm/s	0.07 ± 0.02	0.06 ± 0.02	<0.001
Mitral flow velocity to early mitral annular velocity (*E*/*e*′)	9.4 ± 3.3	13.4 ± 7.5	<0.001
Right ventricular systolic pressure, mmHg	27.3 ± 7.9	31.6 ± 10.7	<0.001
Left ventricular ejection fraction, %	64.0 ± 5.9	63.4 ± 6.3	0.016
HFA-PEFF points			<0.001
0–1 (low probability)	243 (21.6)	61 (4.1)	
2–4 (intermediate probability)	660 (58.7)	494 (33.1)	
5–6 (high probability)	222 (19.7)	937 (62.8)	

Data are presented as the mean ± standard deviation, median (interquartile range), or *n* (%).

AI, artificial intelligence; ECG, electrocardiography; HFA-PEFF, Heart Failure Association Pre-test Assessment, Echocardiography and natriuretic peptide, Functional testing, Final Etiology; HFpEF, heart failure with preserved ejection fraction.

### Discriminative performance of the artificial intelligence-enabled electrocardiogram model

**Figure 2:**
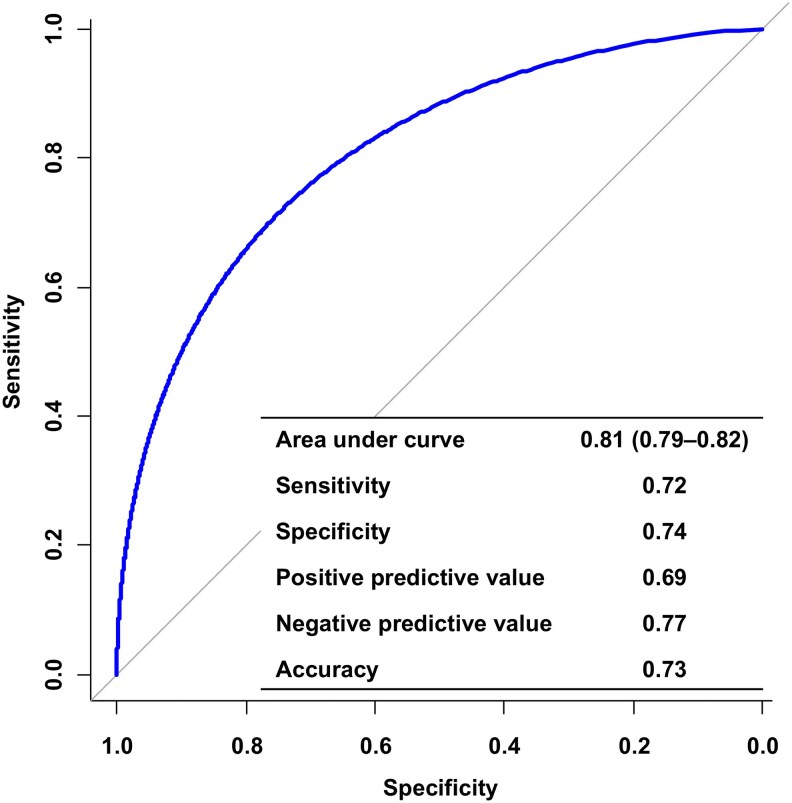
Diagnostic performance of the artificial intelligence-enabled electrocardiogram model. The receiver operating characteristic curve and diagnostic performance of the artificial intelligence-enabled electrocardiogram model for predicting heart failure with preserved ejection fraction.

**Figure 3:**
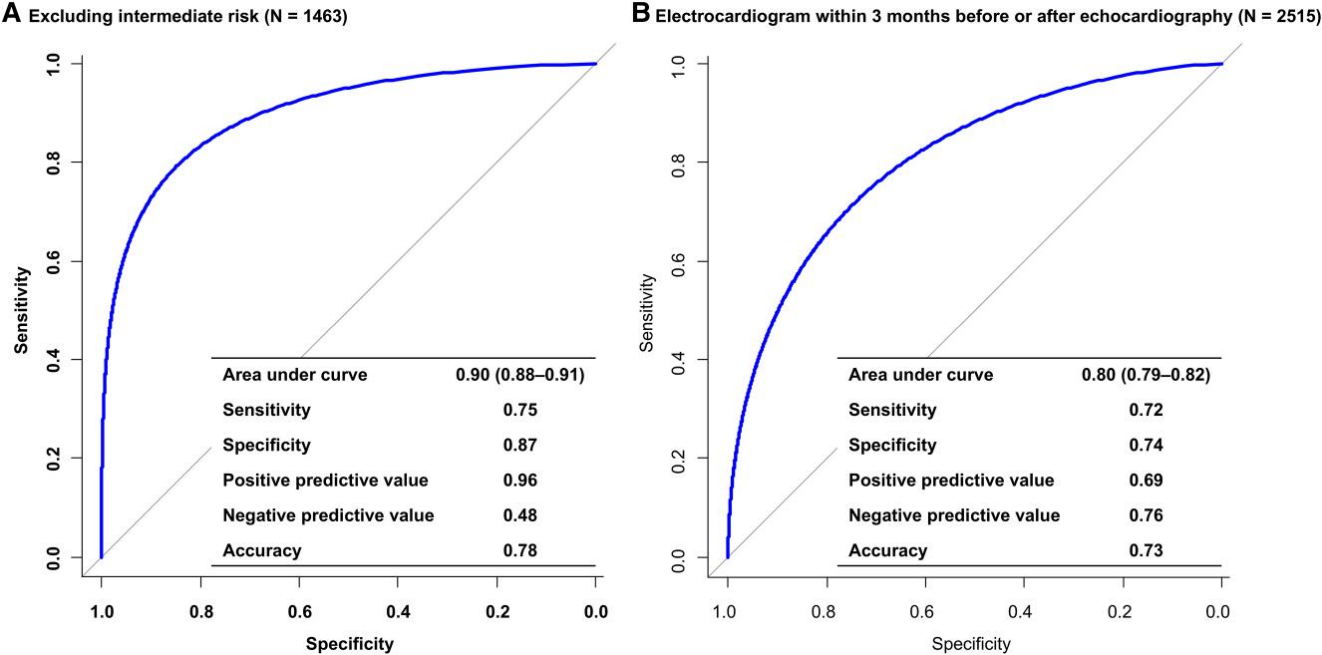
Sensitivity analyses. Receiver operating characteristic curves and diagnostic performance of the artificial intelligence-enabled electrocardiogram model for predicting heart failure with preserved ejection fraction in (*A*) patients excluding those with intermediate risk, and (*B*) those with an electrocardiogram performed within 3 months before or after echocardiography.

**Figure 4:**
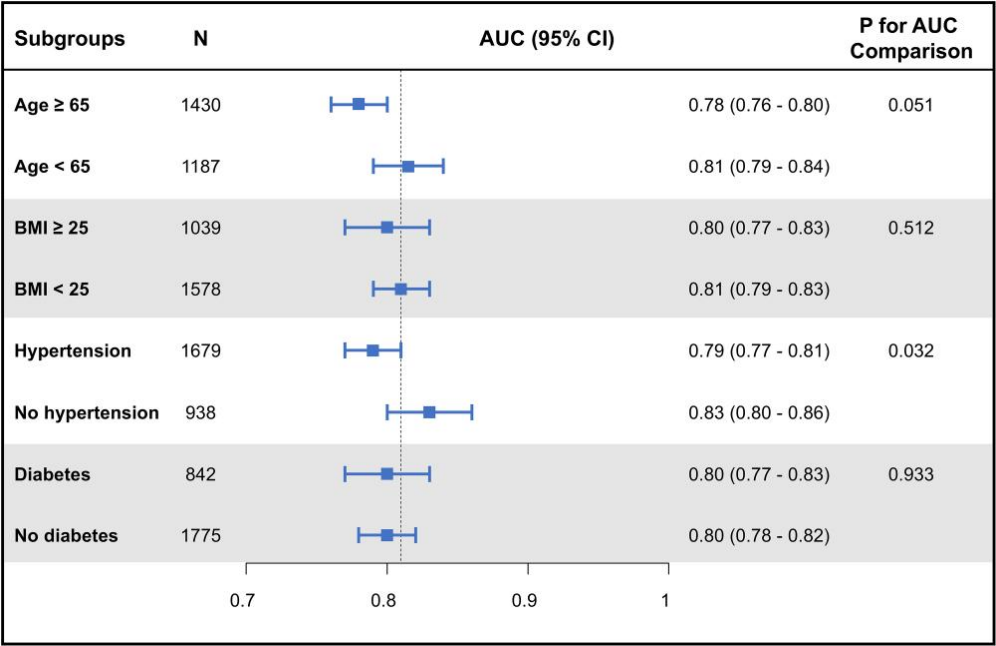
Subgroup analysis. Forest plot displaying the AUC stratified by age, BMI, hypertension, and diabetes mellitus. AUC, area under the curve; BMI, body mass index; CI, confidence interval.

The AI-enabled ECG model demonstrated strong discriminative performance for predicting HFpEF, with an AUC of 0.81 (95% CI 0.79–0.82) in the test set (*Figure 2*). At the Youden index threshold, the model achieved a sensitivity of 0.72, specificity of 0.74, positive predictive value of 0.69, and negative predictive value of 0.77. Sensitivity analyses excluding cases with intermediate risk (AUC 0.90; 95% CI 0.88–0.91) or a time interval greater than 3 months between echocardiography and ECG (AUC 0.80; 95% CI 0.79–0.82) also yielded similar results (*Figure 3*). When performance was evaluated across subgroups stratified by age, body mass index, hypertension, and diabetes mellitus, the model maintained consistent accuracy, with AUC values ranging from 0.78 to 0.83 across all strata (*Figure 4*).

### Clinical outcomes

**Figure 5:**
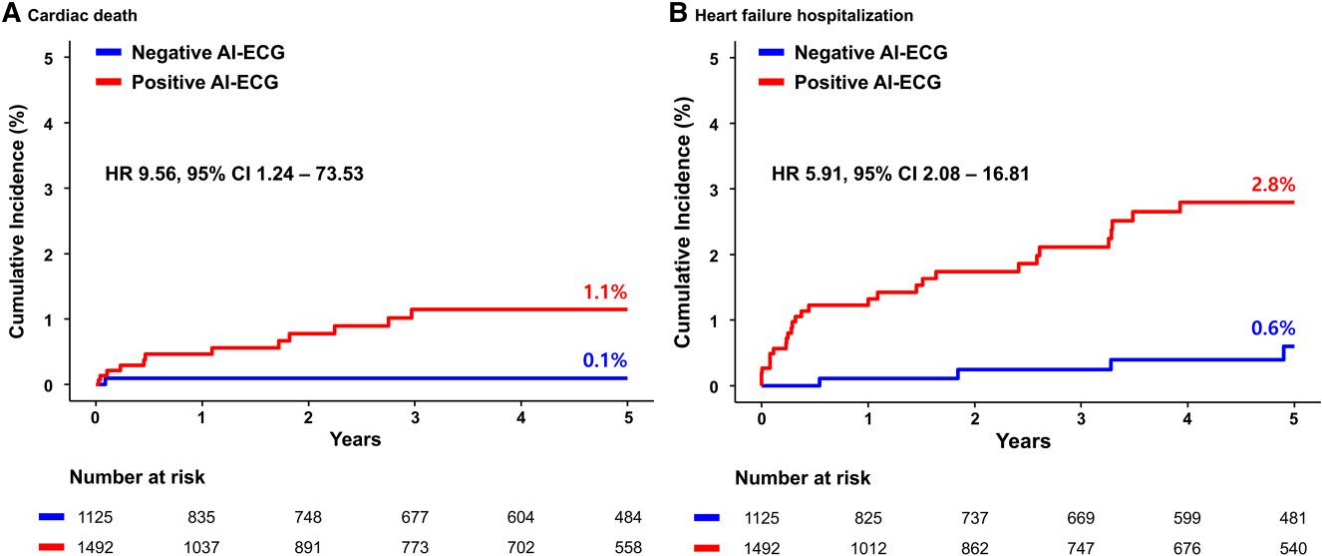
Prognosis according to the artificial intelligence-enabled electrocardiogram model. Kaplan–Meier curves illustrating the cumulative incidences of (*A*) cardiac death and (*B*) heart failure hospitalization, stratified according to positive and negative predictions from the artificial intelligence-enabled electrocardiogram model. AI, artificial intelligence; CI, confidence interval; ECG, electrocardiogram; HR, hazard ratio.

Patients in test cohort were followed up for a median of 4.0 years (interquartile range, 0.8–5.0 years). In the test cohort, the positive AI-ECG group exhibited a higher risk of adverse clinical outcomes compared with the negative AI-ECG group, similar to the increased risk observed in the HFpEF group relative to the control group. Specifically, the positive AI-ECG group had higher risks of cardiac death (1.1% vs. 0.1%; HR 9.56, 95% CI 1.24–73.53; *P* = 0.030), HF hospitalization (2.8% vs. 0.6%; HR 5.91, 95% CI 2.08–16.81; *P* = 0.001), and the composite outcome of both (3.7% vs. 0.7%; HR 6.33, 95% CI 2.50–16.07; *P* < 0.001) (*Table 3* and *Figure 5*). When patients were placed into four groups based on HFpEF vs. control and positive AI-ECG vs. negative AI-ECG classifications, the risk of the composite outcome significantly differed among groups (true positive: 5.3%, false negative: 2.5%, false positive: 0.5%, true negative: 0.2%; log-rank *P* < 0.001) (see Supplementary material online, *Figure S3*).

**Table 3:** Clinical outcomes at 5 year

Outcomes			Hazard ratio (95% CI)	*P*-value
	**Control (*N* = 1458)**	**HFpEF (*N* = 1159)**		
Composite Outcome	4 (0.4)	40 (5.1)	13.37 (4.78–37.38)	<0.001
Cardiac death	2 (0.2)	11 (1.4)	7.18 (1.59–32.42)	0.010
Hospitalization for heart failure	2 (0.2)	31 (4.0)	20.82 (4.98–87.02)	<0.001
	**Negative AI-ECG** (***N* = 1125)**	**Positive AI-ECG** (***N* = 1492)**		
Composite Outcome	5 (0.7)	39 (3.7)	6.33 (2.50–16.07)	<0.001
Cardiac death	1 (0.1)	12 (1.1)	9.56 (1.24–73.53)	0.030
Hospitalization for heart failure	4 (0.6)	29 (2.8)	5.91 (2.08–16.81)	0.001

Cumulative incidence of clinical outcomes is presented as event number (Kaplan–Meier estimates).

AI, artificial intelligence; CI, confidence interval; ECG, electrocardiography; HFpEF, heart failure with preserved ejection fraction.

## Discussion

This study developed an AI-enabled ECG model for predicting HFpEF, using an HFA-PEFF score ≥5 as the reference standard, then assessed its discriminative performance and prognostic relevance in an all-comer population undergoing transthoracic echocardiography. The model demonstrated good discriminative ability (AUC 0.81) for predicting HFpEF, with consistent performance across subgroups stratified according to clinical risk factors for HFpEF. Furthermore, patients with a positive AI-ECG classification had significantly higher risks of cardiovascular death and HF hospitalization relative to patients with a negative AI-ECG classification, demonstrating the prognostic relevance of the model.

### Diagnostic challenges of heart failure with preserved ejection fraction and the need for effective screening tools

HFpEF represents a serious public health challenge, considering its steadily increasing prevalence among the ageing population and individuals with multiple comorbidities, leading to nearly half of all HF cases.^1^ However, HFpEF remains underdiagnosed in routine clinical practice, contributing to as many as one-third of cases of unexplained dyspnoea.^13^ This under-recognition is particularly concerning given recent advancements in HFpEF management that have transformed the therapeutic landscape. Although HFpEF previously was considered a condition with limited or no effective treatments, landmark trials have demonstrated the efficacy of novel therapies such as sodium-glucose cotransporter 2 inhibitors, nonsteroidal mineralocorticoid receptor antagonists, and glucagon-like peptide-1 receptor agonists. These therapies have demonstrated significant reductions in cardiovascular mortality, HF hospitalization, and symptoms among HFpEF patients.^14–17^ These advancements emphasize the need for accurate and timely diagnosis to enable early initiation of evidence-based therapies.

Current diagnostic frameworks for HFpEF, such as the HFA-PEFF and H₂FPEF scores, heavily rely on advanced imaging modalities, including echocardiographic assessments of diastolic dysfunction and structural abnormalities.^5,6^ Although these approaches achieve high diagnostic accuracy, they require substantial resources and cost, creating barriers to widespread implementation. These challenges highlight the urgent need for simple, accessible, and cost-efficient screening tools to address diagnostic gaps. In this context, Reddy *et al*. proposed the HFpEF-ABA score, a simplified model that uses only three clinical variables—age, body mass index, and history of atrial fibrillation—to predict HFpEF.^18^ This model demonstrated good discriminative ability, with an AUC ranging from 0.80 to 0.85. However, its reliance on only three clinical variables limits its capacity to capture the full spectrum of HFpEF pathophysiology; further validation in larger, diverse external cohorts is needed.

### Artificial intelligence-enabled electrocardiogram models for predicting heart failure with preserved ejection fraction

Recent advancements in AI technology have shown promising results in addressing the diagnostic challenges of HFpEF through innovative approaches. One approach involves interpreting echocardiographic images using trained AI algorithms. Tromp *et al*. developed a fully automated deep learning workflow using echocardiographic data from approximately 1500 patients to measure systolic and diastolic parameters, including *E*/*e*′.^19^ This workflow demonstrated good agreement with expert manual measurements of *E*/*e*′ (mean absolute error of 1.8–2.2) and achieved high accuracy in predicting *E*/*e*′ > 13 (AUC of 0.91). Similarly, Akerman *et al*.^20^ used a convolutional neural network model trained on single apical four-chamber echocardiographic images from 6756 patients (2971 with HFpEF and 3785 controls) to detect HFpEF. The model demonstrated excellent discriminative performance, achieving an AUC of 0.91 in an external test cohort; this finding highlighted its potential utility, particularly for screening in centers without expertise regarding comprehensive diastolic assessments.

Efforts to simplify the diagnostic process without requiring echocardiography have also led to the development of AI-enabled ECG models. The strength of ECG lies in its widespread availability, even in primary healthcare settings, making ECG-based AI models suitable for broad population screening. These models leverage the ability of ECG to detect changes in cardiac structure and function associated with HF, which often manifest as electrical conduction abnormalities and alterations in excitation–contraction coupling observable on ECG.^21^ By harnessing advanced AI technologies, these models have the potential to identify early indicators of HFpEF, enabling timely and accurate diagnoses. Kagiyama *et al*.^22^ conducted a multicenter prospective study involving 1202 patients to develop a machine learning model for prediction of *e*′ and left ventricular diastolic dysfunction using clinical and ECG features. The model demonstrated an acceptable correlation and error in estimating *e*′ (Pearson's adjusted *R*^2^ = 0.46; mean absolute error = 1.93). Additionally, it exhibited good discriminative performance (AUC 0.80) for predicting diastolic dysfunction based on the most recent diastolic function guidelines.^10^ Building on this progress, Lee *et al*. utilized data from approximately 300 000 patients with both ECG and echocardiographic data to develop a model capable of predicting increased left ventricular filling pressure and diastolic dysfunction grade using only ECG. This model achieved remarkable accuracy, with an AUC exceeding 0.9.^23^ These findings highlight the potential of AI-enabled ECG models as initial clinical tools for assessing left ventricular diastolic dysfunction.

The present study expands the utility of AI-enabled ECG models by focusing on the prediction of HFpEF using the HFA-PEFF diagnostic algorithm. The HFA-PEFF algorithm integrates NT-proBNP, a biomarker exhibiting high negative predictive value, with echocardiographic parameters. By utilizing the HFA-PEFF algorithm as the gold standard, this study aimed to enable more accurate prediction of HFpEF, surpassing the limited objective of predicting impaired diastolic function on echocardiography. The AI-enabled ECG model developed in this study demonstrated effective discriminative performance in HFpEF identification, with an AUC of 0.81. One limitation of this study is that individuals with an intermediate probability of HFpEF, whose status could not be clearly determined, were included in the control group. While it is likely that some of these individuals do have HFpEF, this approach was chosen to train the model using only definitive HFpEF cases. Notably, when intermediate probability individuals were excluded and only patients whose HFpEF status could be classified using the HFA-PEFF algorithm were included in a sensitivity analysis, the model still demonstrated excellent discriminative performance, with an AUC of 0.90. In addition to its accuracy, the study included a broad population with minimal exclusions, targeting an all-comer cohort, and demonstrated consistent results across subgroups stratified by various risk factors. These findings suggest that the model can serve as an effective screening tool for identifying patients who may benefit from additional advanced assessments, such as echocardiography. Another point that need mention is that the model demonstrated a significant association with HF-related outcomes, such as cardiac death and hospitalization. Notably, patients with a negative AI-ECG classification had only one-fifth the risk of cardiac death relative to those with a positive AI-ECG classification; their absolute incidence of cardiac death was 0.1%. This finding strongly supports the model's suitability as a screening tool. Based on the results of this study, future research should prioritize the development of models with greater accuracy, particularly improved negative predictive value, to optimize their utility as screening tools. An important direction for future research is the development of the model to include patients with atrial fibrillation, a population not specifically addressed in the current analysis. Atrial fibrillation is not only highly prevalent in HFpEF, affecting up to 40% of patients, but also shares common pathophysiological mechanisms with HFpEF.^24,25^ In this context, active, evidence-based management of atrial fibrillation is emphasized in patients with HFpEF.^4^ In the present study, the model was developed using ECG data from patients in sinus rhythm, with atrial fibrillation patients deliberately excluded to enable consistent ECG feature extraction and reliable model training. Building on these findings, future research should focus on developing rhythm-specific models to address the variability introduced by atrial fibrillation. Incorporating this population is both important and necessary to improve the generalizability and clinical applicability of the model. Additionally, it is essential to determine whether the algorithm can genuinely improve patient care in clinical practice.

### Limitations

Some limitations should be acknowledged. First, this study was conducted in a single tertiary care hospital, which may restrict the generalizability of the findings. Therefore, external validation in multicenter settings, particularly in primary care populations, is needed. In particular, it will be important to evaluate the model's performance under real-world conditions, including scenarios involving low-quality ECGs. As such, the significance of this study lies not in its immediate applicability to routine clinical practice, but rather in its potential to support efforts to overcome the diagnostic challenges of HFpEF through emerging technologies. Second, the model was exclusively developed using sinus rhythm ECGs, limiting its relevance to patients with atrial fibrillation, which is a major contributor to HFpEF. Given the high prevalence of atrial fibrillation in HFpEF, this limitation may restrict the clinical applicability of the current model and underscores the need for future models that include patients with atrial fibrillation. Third, patients with an intermediate likelihood according to the HFA-PEFF algorithm were classified as controls in this study, but non-invasive stress tests or invasive diagnostics are recommended for definitive diagnosis. Although this approach facilitates a clearer characterization of HFpEF features, it may increase the risk of false negative classification. Fourth, this study included an all-comer population undergoing echocardiography without detailed information on symptoms. Although only patients with NT-proBNP measurements were included, indicating a likely evaluation for suspected HF, NT-proBNP can also be elevated due to non-cardiac causes or in the absence of symptoms. Therefore, it is important to acknowledge that the HFA-PEFF algorithm is probabilistic in nature and cannot provide a definitive diagnosis of HFpEF without appropriate clinical context. Additionally, some asymptomatic patients with ACC/AHA Stage B HF may have been included.^26^ Given these limitations and the aim of the study, it is important to clarify that the significance of this work lies not in establishing the AI-ECG model as a tool for definitive diagnosis of HFpEF, but in its potential to simplify the diagnostic process and support earlier detection. Fifth, this study lacks model interpretability, which is a common limitation of AI known as the ‘black box’ phenomenon. As a result, it remains unclear which ECG features most strongly influenced the model's predictions.

## Conclusions

The AI-ECG model is a reliable tool for predicting HFpEF, as defined by the HFA-PEFF score, and effectively stratifies patients with worse prognoses. Integration of the AI-ECG model into clinical practice has the potential to simplify and enhance the diagnostic process for HFpEF.

## Supplementary Material

ztaf080_Supplementary_Data

## Data Availability

Data that support the findings of this study are available from the corresponding author upon reasonable request.
