# Supplementary material for: Artificial intelligence-enabled electrocardiogram model for predicting heart failure with preserved ejection fraction: a single-center study
Source: Eur Heart J Digit Health. 2025 Jul 17;6(5):959–68. doi: 10.1093/ehjdh/ztaf080 (PMC12450526; doi:10.1093/ehjdh/ztaf080)
Supplement: ztaf080_Supplementary_Data [file ztaf080_supplementary_data.docx]

**Supplemental Appendix**

**Artificial Intelligence-Enabled Electrocardiogram Model for Predicting Heart Failure with Preserved Ejection Fraction – A Single-Center Study**

**Table of Contents**

- **Supplementary Figures and Figure Legends**
- **Supplementary Tables**

**Supplementary Figures and Figure Legends**

**Figure S1. Schematic Representation**

**
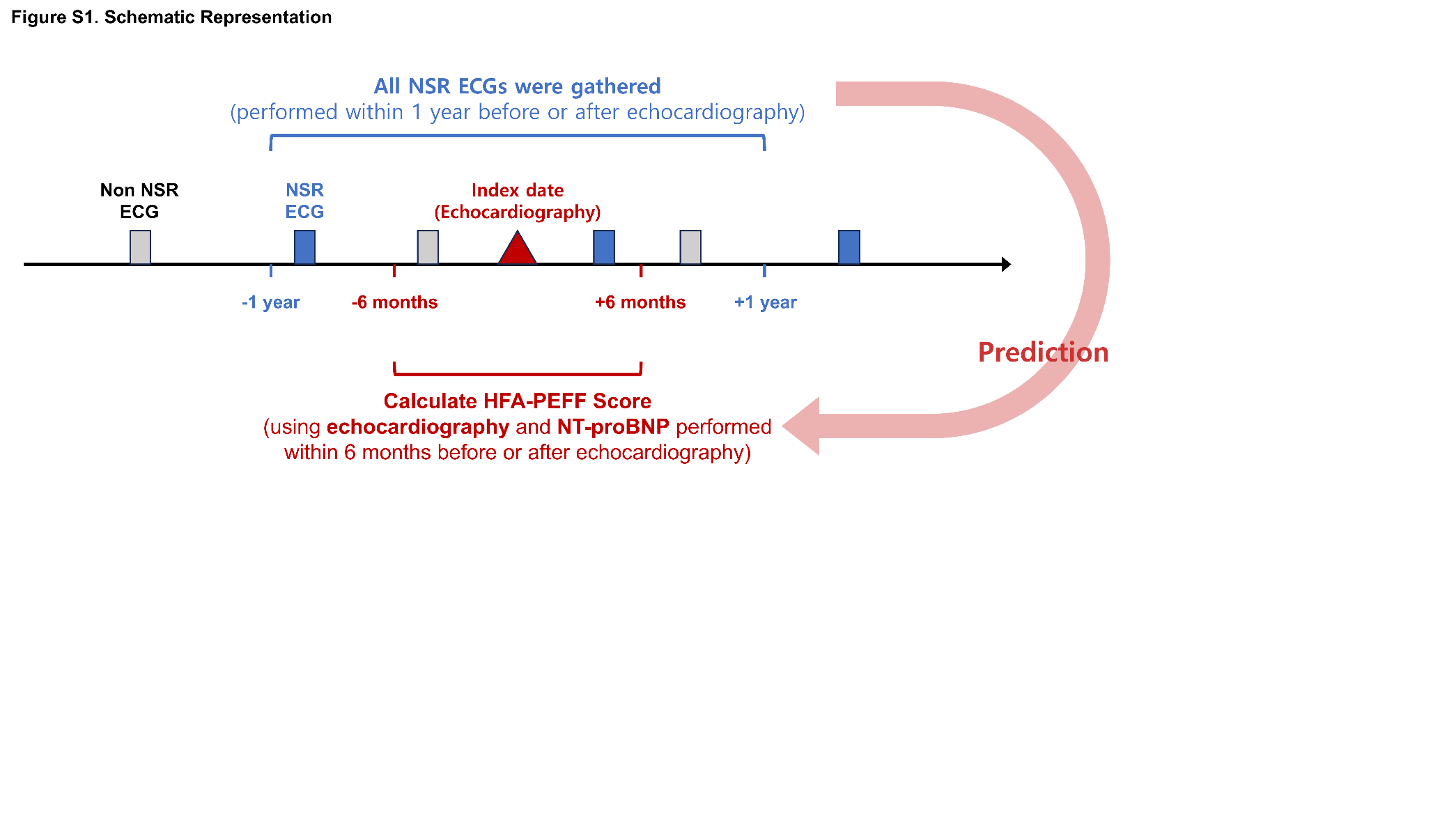
**

The HFA-PEFF score was calculated using echocardiography and NT-proBNP performed within 6 months before or after echocardiography. Using NSR ECGs performed within 1 year before or after echocardiography, an artificial intelligence-enabled ECG model was developed to predictor HFpEF, defined as the HFA-PEFF score ≥5.

Abbreviations: ECG, electrocardiogram; HFA-PEFF, Heart Failure Association Pre-test Assessment, Echocardiography and natriuretic peptide, Functional testing, Final Etiology; NSR, normal sinus rhythm; NT-proBNP, N-terminal prohormone of B-type natriuretic peptide.

**Figure S2. Architecture of the Artificial Intelligence-Enabled Electrocardiogram Model for HFpEF Prediction**

**
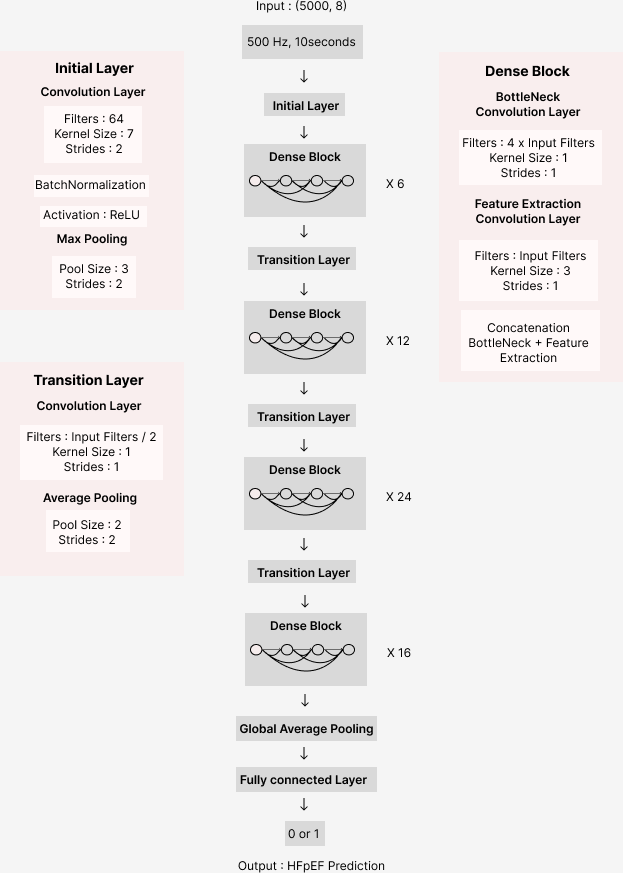
**

Abbreviations: HFpEF, heart failure with preserved ejection fraction; ReLU, rectified linear unit.

**Figure S3. Cardiac Death or Hospitalization for Heart Failure According to HFpEF Probability and Artificial Intelligence-Enabled Electrocardiogram Model**

**
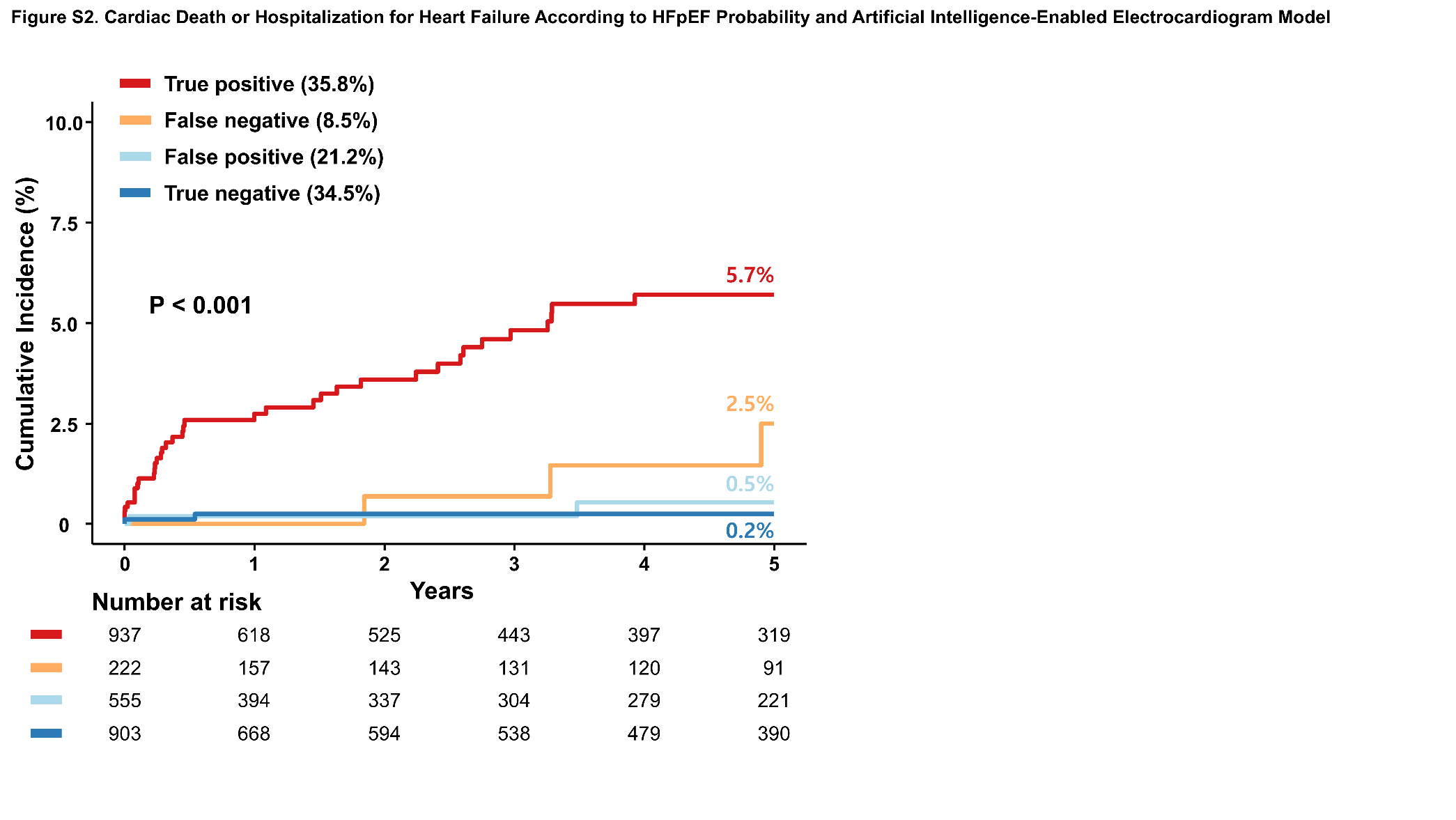
**

The Kaplan-Meier curves illustrate the cumulative incidence of the composite outcome of cardiac death and heart failure hospitalization, stratified by HFpEF vs. control and positive vs. negative artificial intelligence-enabled electrocardiogram model prediction.

Abbreviations: HFpEF, heart failure with preserved ejection fraction.

**Supplementary Tables**

**Table S1. Baseline Characteristics Across Datasets**

| **Variables** | **Training**  **N = 9,156** | **Validation**  **N = 1,308** | **Test**  **N = 2,617** | **P value** |
| --- | --- | --- | --- | --- |
| Age | 63.5 ± 13.7 | 64.1 ± 14.0 | 63.9 ± 13.5 | 0.135 |
| Male | 4,981 (54.4) | 717 (54.8) | 1,423 (54.4) | 0.959 |
| Systolic blood pressure, mmHg | 125.7 ± 19.4 | 126.2 ± 19.0 | 126.1 ± 19.8 | 0.491 |
| Diastolic blood pressure, mmHg | 71.2 ± 12.8 | 71.1 ± 12.9 | 71.3 ± 13.1 | 0.893 |
| Body mass index, kg/m^2^ | 24.4 ± 3.8 | 24.4 ± 3.8 | 24.3 ± 3.7 | 0.234 |
| Hypertension | 5,717 (62.4) | 845 (64.6) | 1,679 (64.2) | 0.124 |
| Diabetes mellitus | 2,851 (31.1) | 399 (30.5) | 842 (32.2) | 0.490 |
| Atrial fibrillation | 239 (2.6) | 23 (1.8) | 55 (2.1) | 0.084 |
| Chronic kidney disease | 470 (5.1) | 69 (5.3) | 133 (5.1) | 0.967 |
| Current or past smoking | 2,754 (30.1) | 375 (28.7) | 771 (29.5) | 0.527 |
| History of percutaneous coronary intervention | 597 (6.5) | 97 (7.4) | 186 (7.1) | 0.330 |
| History of myocardial infarction | 389 (4.2) | 46 (3.5) | 108 (4.1) | 0.462 |
| Chronic obstructive lung disease | 495 (5.4) | 66 (5.0) | 152 (5.8) | 0.577 |
| N-terminal prohormone of B-type natriuretic peptide | 172.1 [59.1-604.7] | 165.5 [56.6-589.0] | 174.8 [59.9-644.4] | 0.480 |
| ***Echocardiography*** |  |  |  |  |
| Left ventricular internal diameter in diastole, mm | 49.4 ± 5.6 | 49.3 ± 5.6 | 49.3 ± 5.6 | 0.617 |
| Left ventricular internal diameter in systole, mm | 29.6 ± 4.7 | 29.5 ± 4.5 | 29.6 ± 4.8 | 0.906 |
| Left atrial volume index, mL/m^2^ | 40.0 ± 18.4 | 39.0 ± 18.1 | 39.6 ± 20.9 | 0.189 |
| Left ventricular mass index, g/m^2^ | 110.1 ± 35.3 | 109.3 ± 34.6 | 110.6 ± 37.9 | 0.530 |
| Peak early diastolic mitral flow velocity (E), m/s | 0.71 ± 0.29 | 0.70 ± 0.28 | 0.70 ± 0.29 | 0.149 |
| Peak late diastolic mitral flow velocity (A), m/s | 0.78 ± 0.25 | 0.79 ± 0.24 | 0.78 ± 0.25 | 0.719 |
| Early to late diastolic mitral flow velocities (E/A) | 0.98 ± 0.99 | 0.94 ± 0.50 | 0.96 ± 0.59 | 0.265 |
| Peak early diastolic velocity of mitral annulus (e’), cm/s | 0.07 ± 0.02 | 0.07 ± 0.02 | 0.07 ± 0.02 | 0.865 |
| Mitral flow velocity to early mitral annular velocity (E/e’) | 11.9 ± 6.6 | 11.7 ± 7.0 | 11.7 ± 6.4 | 0.265 |
| Right ventricular systolic pressure, mmHg | 30.1 ± 10.6 | 29.9 ± 9.9 | 29.8 ± 9.9 | 0.631 |
| Left ventricular ejection fraction, % | 63.8 ± 6.1 | 63.8 ± 5.9 | 63.7 ± 6.2 | 0.644 |
| HFA-PEFF points |  |  |  | 0.942 |
| 0 – 1 (low probability) | 1,074 (11.7) | 163 (12.5) | 304 (11.6) |  |
| 2 – 4 (intermediate probability) | 4,026 (44.0) | 565 (43.2) | 1,154 (44.1) |  |
| 5 – 6 (high probability) | 4,056 (44.3) | 580 (44.3) | 1,159 (44.3) |  |

Data are presented as the mean ± standard deviation, median [interquartile range], or n (%).

Abbreviations: HFA-PEFF, Heart Failure Association Pre-test Assessment, Echocardiography and natriuretic peptide, Functional testing, Final Etiology; HFpEF, heart failure with preserved ejection fraction.
